# Diagnosing Vitreoretinal Lymphomas—An Analysis of the Sensitivity of Existing Tools

**DOI:** 10.3390/cancers14030598

**Published:** 2022-01-25

**Authors:** Anahita Sehgal, Jose S. Pulido, Arman Mashayekhi, Tatyana Milman, Gabor Gy Deák

**Affiliations:** 1 Department of Ophthalmology, Wills Eye Hospital, Sidney Kimmel Medical College, Thomas Jefferson University, Philadelphia, PA 19107, USA; Anahita.Sehgal@students.jefferson.edu (A.S.); jPulido@willseye.org (J.S.P.); 2 Bower Laboratory for Translational Medicine Vickie and Jack Farber, Vision Research Center, Wills Eye Hospital, Philadelphia, PA 19107, USA; 3 Department of Ophthalmology, Mayo Clinic, Jacksonville, FL 32224, USA; arman_mash@yahoo.com; 4 Department of Pathology, Wills Eye Hospital, Sidney Kimmel Medical College, Thomas Jefferson University, Philadelphia, PA 19107, USA; tmilman@willseye.org; 5 Department of Ophthalmology, Medical University of Vienna, 1090 Vienna, Austria

**Keywords:** vitreoretinal lymphoma, primary vitreoretinal lymphoma (PVRL), diagnostics, cytology, flow cytometry, MyD88, PCR, IgH rearrangement, cytokine, IL10, IL6

## Abstract

**Simple Summary:**

Diagnostics of vitreoretinal lymphoma is very challenging, as the possibility of receiving false negative results is common. We retrospectively analyzed the sensitivity of the most commonly used diagnostic methods including ancillary immunohistochemistry, Myeloid Differentiation Factor 88 (MyD88) L256P mutation analysis, polymerase chain reaction (PCR) for monoclonal rearrangements of immunoglobulin heavy chain (IgH) and T-cell Receptor (TCR) genes, flow cytometry, and IL10 and IL6 analysis, to diagnose vitreoretinal lymphomas from published data in the literature. MyD88 mutation analysis caused by a hotspot mutation in MyD88 was the most sensitive and had the lowest coefficient of variation.

**Abstract:**

Vitreoretinal lymphoma (VRL) is a rare ocular pathology that is notorious for mimicking chronic uveitis, which is a seemingly benign condition in comparison. The most common form of VRL is the diffuse large B-cell type, and there has been a high mortality rate. This dismal prognosis can be improved significantly if the disease is diagnosed early, but until now there is no consensus on an appropriate diagnostic algorithm. We conducted a retrospective search of PubMed Central^®^ and analyzed results from thirty-three studies that were published between 2011–2021. The chosen studies incorporated some popular testing tools for VRL, and our analyses focused on comparing the average sensitivity of five diagnostic methods. The methods included cytology including ancillary immunohistochemistry, Myeloid Differentiation Factor 88 (MyD88) mutation analysis, polymerase chain reaction (PCR) for monoclonal rearrangements of immunoglobulin heavy chain (IgH) and T-cell Receptor (TCR) genes, flow cytometry, and IL10 and IL6 analysis. Across the varied diagnostic methods employed in thirty-three studies explored in this analysis, MyD88 mutation assay emerged as a strong contender given its sensitivity and low coefficient of variation. There is an imminent need for the introduction of newer assays that can further improve the sensitivity of identifying MyD88 mutation in cancer cells seen in the vitreous.

## 1. Introduction

**Figure 1:**
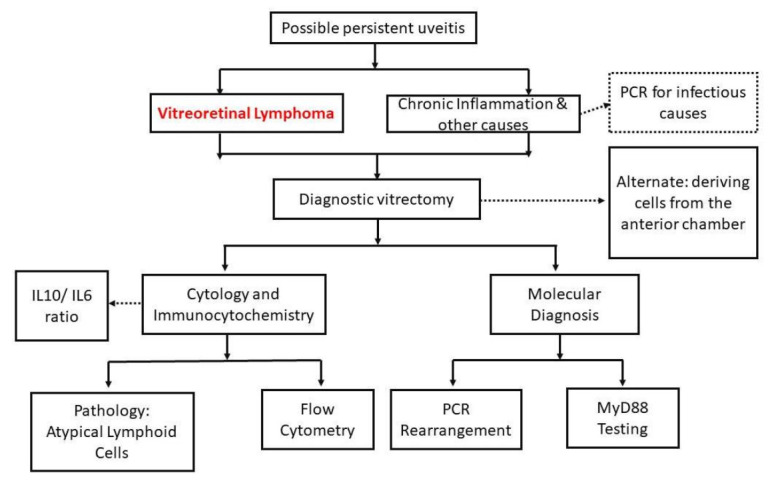
Flowchart representing the different diagnostic options that exist to home in on a potential VRL diagnosis.

Vitreoretinal Lymphoma presents a difficult challenge for clinicians and pathologists alike. Diffuse large B-cell lymphoma (DLBCL) is the most commonly seen vitreoretinal lymphoma and is often confused for chronic uveitis (ocular inflammation) [1]. Given its deceptive similarity to uveitis and its association with central nervous system (CNS) involvement, a delay in diagnosis can be a major setback considering the 1–2 year median survival rate [1]. There is a need to shorten the time between onset of symptoms and the appropriate diagnosis. In fact, a recent study conducted at Wills Eye Hospital highlighted that the mean duration between onset of symptoms and referral to the Oncology Service was 17 months whereas the median time duration was 8 months [2]. In the literature, various laboratory-based methods have been described to improve the diagnostic accuracy of this notorious masquerader [3,4,5,6,7]. These include cellular-based and molecular methods depicted in Figure 1. We performed a review of the literature to better determine which of the commonly used methods has the highest sensitivity, and therefore can function as a reliable tool in making the correct diagnosis.

## 2. Materials and Methods

### 2.1. Study Design

We conducted a retrospective search of PubMed Central^®^ (PMC) and focused on studies published in the past decade, dated from 1 January 2011 to 31 September 2020 (including ones published online ahead of print). The following keywords were used—vitreoretinal lymphoma, retinal lymphoma, vitreous lymphoma, intraocular lymphoma. We chose to limit our search to since 2011 because molecular methods have only been more widely used within the last decade. Studies included in this review consisted of three or more cases of VRL. Each study comprised an analysis of the efficacy of two or more commonly used diagnostic methods for VRL. The methods included cytology including ancillary immunohistochemistry, Myeloid Differentiation Factor 88 (MyD88) mutation analysis, polymerase chain reaction (PCR) for monoclonal rearrangements of immunoglobulin heavy chain (IgH) and T-cell Receptor (TCR) genes, flow cytometry, and IL10 and IL6 analysis. These selection criteria were collectively and independently determined by three authors (JSP, GGD and AM). Studies that applied only one diagnostic method, as well as systematic reviews and meta-analyses, were excluded.

### 2.2. Data Collection and Bias Assessment

The authors reviewed each study to collate data on the specific diagnostic test used in the study and the number of positive results the test indicated with respect to the total number of positive cases listed. A patient qualified as a positive case if they were enrolled in the study with a final diagnosis of VRL. The key findings from each study are summarized in Table 1 and Tables S1–S3. Furthermore, these findings were independently reviewed by another author (AS). Each study had its own potential biases, and confounding conclusions were discussed within the authors and resolved by consensus.

### 2.3. Data Synthesis and Analysis

In this review, we evaluated the accuracy of the diagnostic methods used in each study relative to the number of positive cases identified in the study. In Tables S1–S3, we summarize the number of positive results pertaining to each diagnostic test, the number of positive cases identified, the percent positive of sum and the sensitivity. Our main goal was to average the various sensitivity values obtained per study, and consequently determine the method that has the leading efficacy given variability in data.

Our data analyses encompassed both the mean and the weighted mean, considering each study comprised a different number of positive cases, and we aimed to give the sensitivity values obtained from studies that incorporate an increased number of cases greater significance. We attempted to adjust for the vast variability prevalent in our data by calculating the standard deviation, weighted standard deviation, coefficient of variation (CV) and 95% confidence intervals (CI).

These calculations were performed using Microsoft Excel, software version 16.53 for Mac.

## 3. Results

A total of two hundred and sixty-nine studies were identified through PubMed, and thirty-three studies met with the defined selection criteria. All the studies included were retrospective studies (Table 1).

Table 2 and Table 3 illustrate analyses of the aggregated data specific to each of the diagnostic tests evaluated in this meta-analysis.

Our statistical analyses present the aggregated data in two distinct forms. Table 2 uses a non-weighted average and indicates that checking for the presence of MyD88 and cytokine analyses have the highest sensitivity ([M = 75%, SD = 14%], [M = 86%, SD = 15%]), respectively. Both diagnostic modalities also have the lowest coefficient of variation (0.19 and 0.17, respectively), a unitless measure that speaks to the variance of data independent of the standard deviation. Cytology, PCR IgH gene rearrangement and flow cytometry are shown to be relatively less sensitive in our aggregated data set, and the significance of their decreased sensitivity is established by their increased CVs, especially in comparison to MyD88 testing and cytokine analyses. Elevated CVs for these testing methods indicate the heightened variability in the data collected across studies, suggesting that while some studies established these testing modalities as effective, others did not reproduce the accuracy associated with these modalities.

Table 3 however highlights a different statistical perspective. When the weighted average is calculated, checks for the presence of MyD88 and PCR rearrangement are shown to have the highest sensitivity ([wM = 74%, wSD = 11%], [wM = 80%, wSD = 26%]), respectively. Although MyD88 has increased accuracy across both tables, Table 3 shows that PCR rearrangement has a higher sensitivity compared to Table 2. This difference can be attributed to difference in sample size amongst the thirty studies included in this metanalysis. Consequently, PCR rearrangement was more frequently used in studies with larger sample sizes but there was a high CV showing that there is a difference in the use between centers. Cytokine analyses have a relatively lower weighted mean, suggesting that it was not used as often as PCR rearrangement but in the selected studies it was used, the results were accurate and precise. This is further substantiated by the lower CV of cytokine analyses compared to PCR rearrangement. Cytology and Flow cytometry continued to show relatively less sensitivity ([wM = 61%, wSD = 27%], [wM = 64%, wSD = 24%]), respectively. When evaluating based on weighted average, flow cytometry shows better accuracy compared to cytology, which shows a decrease relative to its mean in Table 2.

## 4. Discussion

The average time for diagnosis of primary vitreoretinal lymphoma (PVRL) after symptom onset is 1 year, and patients will continue to succumb to the disease (primary or secondary) unless we improve our diagnostic methods [1]. Our metanalysis highlights the calculated sensitivity averages of five of the most used diagnostic tools across thirty-three studies over the past decade. Although each method presents with its own advantages and disadvantages, statistically, checking for the presence of MyD88 mutations has maintained its high sensitivity (M = 75% ± 14%; wM = 74% ± 11%) across both forms of analyses conducted in our study. We chose to calculate the mean to provide a standard of comparison across the success rate of each diagnostic test implicated, whereas our decision to also calculate the weighted mean came from an understanding of providing studies with larger sample sizes greater significance. A comparison of both the mean and the weighted mean allows us to interpret the strength of each method with and without consideration of sample size; a large variation between the two values for a specific method would lead us to believe that some methods that are used more frequently in the studies with a larger sample size may not be as accurate or precise. MyD88 testing also showed a consistently low CV, this highlights that there was minimal discrepancy among different studies regarding the reliability of the test to reproduce true positives.

A difference of ~0.10 is highlighted between the mean and the weighted mean for cytology. The confidence intervals calculated suggested that this variation is likely not arbitrary but significant for a difference in accuracy of method. Considering the wM [61% ± 27%] is lower than the M [71% ± 28%], it can be argued that cytology did not produce a high degree of true positives in the studies with considerably large sample sizes that researched the success of this method.

Historically, cytological examination has been the gold standard for diagnosing VRL from a vitreous sample [41]. However, the success of this method requires a highly experienced ocular pathologist. Vitreous aspirates are frequently paucicellular due to limited involvement of the vitreous by lymphoma or as a consequence of prior steroid therapy. Additionally, large B cell lymphoma cells are notoriously fragile and apoptotic, contributing to the diagnostic challenge. Furthermore, vitreous fluid in vitreoretinal lymphoma frequently contains a dominant infiltrate of chronic inflammatory cells (T cells and macrophages), which obscure the rare and degenerated malignant lymphocytes. The low-level involvement of the vitreous fluid by lymphoma and frequent apoptosis of the lymphoma cells contribute not only to the challenges of cytomorphologic assessment, but also to the challenges of immunohistochemical ancillary studies for lymphoma diagnosis and typing.

Flow cytometry and FACS (florescence activated cell sorting) can be used concomitantly to identify a clonal B cell population, assessing the cell size and clonal expression of surface antigens, such as kappa or lambda light chain restriction [1]. Although flow cytometry methodology has evolved in the past decades, it continues to require a significant number of intact, viable cells for diagnosis (10^5^–10^6^ cells) [42]. Thus, suboptimal cellularity and viability are frequent limitations of flow cytometry in its ability to diagnose PVRL.

Cytokine analyses are emerging as another popular diagnostic study, and our calculations show that this method is also supported with high sensitivity values (M = 86% ± 15%; wM = 73% ± 17%). Interleukin 10 has been established to have an anti-inflammatory effect on the retina, and thus decreases leakage through the blood retinal barrier similar to its role in the nervous system [1]. Previously, studies have argued for the likelihood of lymphomas secreting cytokines such as IL10 to prevent their recognition by the immune system, especially considering that the cells have honed to the vitreous only because it is protected from the immune system [1]. A caveat to this diagnostic test is that IL10 is elevated both in the vitreous and the aqueous of the eye in patients with VRL [1,43]. Conversely, Raja et al. indicated that elevated IL6 levels did not demonstrate any statistically meaningful difference between patients with lymphoma and those with uveitis [43].

Most experts advocate for the use of IL10:IL6 ratio >1 as indirect evidence supporting the VRL diagnosis [7]. This method however cannot concretely identify VRL independently, because the ELISA (enzyme-linked immunosorbent assay) as well as Luminex assay levels used to derive the ratio differ between laboratories as well as between one “kit” and another from the same company/laboratory [1]. There also exists variability of “normal” levels, and therefore a difference such as an increase in IL10 or IL6 is most helpful in following treatment progression. However, the merits of using indirect evidence are unarguable, and an improvement in IL level assessment techniques in the future could increase the reliability of this test as an independent diagnostic tool [43,44,45,46,47,48,49]. Furthermore, some medical oncology services do not accept the IL10/IL6 as definitive diagnosis but only as confirmative.

Amidst these diagnostic tests, MyD88 L265P mutation analysis had high sensitivity and a low coefficient of variation. MyD88 is a signaling protein derived from a driver gene found in hematologic B cell malignancies. The L265P missense mutation is thought to promote cancer cell survival and is enriched in lymphoplasmacytic lymphomas—especially in Waldenström macroglobulinemia and lymphomas of immune-privileged sites, including VRL/CNS lymphomas [32,50]. Prior studies have demonstrated that MyD88 mutation analysis has a sensitivity that ranges between 69% and 88% in the diagnosis of PVRL [31,32,33,51]. It is worthwhile to note that MyD88 mutation will not detect T cell lymphomas, a small subset of PVRL/ CNS DLBCL, and some B cell lymphomas of other types. Additionally, identification of MyD88 mutation may not unequivocally be indicative of PVRL/CNS lymphoma, as lymphomas of other sites with secondary vitreous involvement may also harbor this mutation, so evaluation for systemic diffuse large B cell lymphoma elsewhere in the body is needed upon initial staging [2]. However, considering that the DLBCL VRL is the most common type of VRL with a prevalence (95%), MyD88 mutation analysis has a high sensitivity for the majority of patients with VRL [1]. In addition to its diagnostic value, MyD88 mutation status is more cost effective and has a shorter turnaround time when compared to the established IgH PCR-based assays adapted by pathology laboratories [1]. The PCR-based IgH rearrangement assay requires a larger quantity of cells and generally has a higher limit of detection (10–20% of clonal B cell population) when compared to MyD88 mutation analysis, which can detect 5% or less mutant cells [51,52]. PCR for IgH and TCR gene rearrangements can also rarely yield a false positive result because of non-uniform (skewed) amplification of gene rearrangements. In the studies reviewed, PCR for IgH rearrangement was found to have a slightly higher sensitivity than MyD88 PCR mutation analysis, but it also has a larger standard deviation and CV, which adds to more uncertainty in the results and its use.

MyD88 mutation analysis is usually performed on a sample obtained from the vitreous or subretinal infiltrates using standard vitrectomy techniques as described by Pulido and coworkers or, more recently, on aqueous samples [1,19,21,53]. The sample is preserved in appropriate preservative (usually Roswell Park Memorial Institute solution) and submitted immediately for an allele-specific polymerase chain reaction for detection of MyD88 L265P mutation [19,21,33,54]. This technique has been effective in detecting MyD88 L265P mutation in undiluted and diluted vitreous specimens and in the aqueous fluid [21]. Detection of MyD88 L265 mutation in ocular samples is based on polymerase chain reaction (PCR) techniques such as droplet digital polymerase chain reaction and amplification-refractory mutation polymerase chain reaction [1,19,21]. Narasimhan and coworkers have used PCR-based sequencing and restriction approach for their cases, and Bonzheim and coworkers have used melting curve analysis of amplification products for detection of MyD88 mutation in their study [32,54].

Recently, several investigators have reported MyD mutation analysis on aqueous samples obtained by paracentesis. This technique is less invasive than pars plana vitrectomy and can be easily performed in the office at the slit lamp using topical anesthesia and can yield about 0.3 mL aqueous for analysis [19,21,53]. In a study by Hiemcke-Jiwa and coworkers, using droplet digital polymerase chain reaction (ddPCR), the investigators were able to detect MyD88 L265P mutation in the aqueous fluid of eight of the nine patients (89%) that had the same mutation in their vitreous fluid samples. The investigators found a sensitivity of 75% (95% CI, 50–92%), specificity of 100%, and a positive predictive value of 100% for vitreous samples. For aqueous samples, sensitivity was 67% (95% CI, 42–92%) with a specificity of 100% and positive predictive value of 100% [21]. In another study, Miserocchi and coworkers reported the results for MyD88 L265 mutation in eight patients with bilateral cytologically proven vitreoretinal lymphoma. Using a modified amplification-refractory mutation polymerase chain reaction approach, the researchers looked for MyD88 mutation in the aqueous of eight patients (15 eyes) and in the vitreous of six patients (8 eyes). Vitreous examination for MyD88 mutation was positive in all tested samples and was consistent with cytological examination in all cases but one. MyD88 L265 mutation was present in six of eight patients (75%). The results of MyD88 L265P mutation in the aqueous and vitreous samples were consistent in seven of the eight eyes with available samples [19]. In a recent case report, Choi and coworkers have documented disappearance of MyD88 L265P mutation from the aqueous fluid of a patient with bilateral vitreoretinal lymphoma after four weekly injections in the non-vitrectomized eye and after two weekly injections in the vitrectomized eye of the patient [53].

A recent publication, which addresses a new technology that allows for the isolation of single B cells from the vitreous, ultimately aims to increase the speed of molecular diagnostic methods for VRL. Although this technology exhibits vast potential, there is still much improvement that needs to occur considering the low sensitivity and specificity of the presence of MyD88 mutation (35% ± 31.3%) found using this method [55]. We believe that such technologies will prove beneficial against the masquerader VRL in the near future but still need some refinement with respect to accuracy.

## 5. Conclusions

Our analyses have shown that each of the five diagnostic methods have their merits, some more sensitive than the others. Given the complicated nature of this disease, it is not wise to expect one method to be enough to establish a diagnosis. MyD88 mutation analysis has emerged as a powerful ancillary study in diagnosis of VRL, with a sensitivity of 69–88%, based on prior studies. Detection of MyD88 mutation supports the diagnosis of a B cell lymphoma, regardless of whether this is PVRL/CNS DLBCL or any other B cell lymphoma, and the presence of mutation essentially rules out uveitis [21]. Since MyD88 mutation is highly enriched in VRL, identification of this mutation can serve as a surrogate marker of B cell clonality (similar to IgH PCR and flow cytometry). Additionally, identification of MyD88 mutation is highly suggestive of PVRL/CNS lymphoma, although it does not exclude other types of B cell lymphoma. Finally, recent studies have demonstrated that MyD88 mutation analysis may serve as a tool for detection of recurrence and monitoring of treatment.

Our study has several important limitations. Although we were able to weigh the studies based on the sample size, we could not stratify the data based on a number of factors that might heavily influence the results of each examination method, such as variability in clinical presentation, amount and quality of the diagnostic material obtained, the stage of the disease, and time since last steroid use. Despite these limitations, our results suggest that MyD88 mutation analysis is a powerful diagnostic method for PVRL, which can serve as an adjunct to cytology.

## Figures and Tables

**Table 1:** Key design features of the studies assessed.

S/N	Study	Study Design	Period of Study	Origin	Patient (N)	Control Group	Number of Controls
1	Giufrrè (2021); Ocul Immunol and Inflamm [8]	Retrospective	2014–2019	Italy	31 patients	NA	NA
2	Tsubota (2020); J Clin Med [9]	Retrospective	2020	Japan	40 patients	NA	NA
3	Arai (2020); Cancer Sci [10]	Retrospective	2020	Japan	10 patients	NA	NA
4	Lee (2020); Haematologica [11]	Prospective	2018–2019	Korea	9 patients	NA	NA
5	Marchese (2019); Br J opthalmol [12]	Retrospective	2016–2018	Italy	13 patients	NA	NA
6	Shi (2021); Ocul Immunol Inflamm [13]	Prospective	2011–2018	China	26 patients	NA	NA
7	Tan (2019); Blood [14]	Prospective	2019	Singapore	3 patients	Patients with chronic inflammation	3
8	Quintyn (2019); Cytopathology [15]	Retrospective	2010–2017	France	15 patients	NA	NA
9	Ito (2019); Graefe’s Archive for Clinical and Experimental Ophthalmology [16]	Retrospective	2001–2016	Japan	39 patients	NA	NA
10	Hoog (2019); Acta Ophthalmol [17]	Prospective	2012–2015	Netherlands	53 patients	NA	NA
11	Yonese (2019); Eur J Haematol [18]	Retrospective	2007–2016	Japan	17 patients	Patients with uveitis	6 patients
12	Miserocchi (2019); Retina [19]	Prospective	2016–2017	Italy	8 patients	NA	NA
13	Carreno (2019); Acta Ophthalmol [20]	Prospective	2018	United Kingdom	18 patients	NA	NA
14	Hiemcke-Jiwa (2018); JAMA Ophthal [21]	Retrospective	2005–2017	Netherlands	63 individuals	NA	NA
15	Nakahara (2018); BMC Opthalmol [22]	Retrospective	2009–2013	Japan	5 patients	NA	NA
16	Lee (2019); Retina [23]	Retrospective	2013–2017	South Korea	43 patients	NA	NA
17	Cho (2018); Ocul Immunol Inflamm [24]	Retrospective	2000–2014	South Korea	53 patients	NA	NA
18	Pochat-Cotilloux (2018); Retina [25]	Retrospective	2009–2014	France	16 patients	Patients with uveitis	103 patients
19	Cani (2017); Oncotarget [26]	Retrospective	2017	United States	4 patients	NA	NA
20	Cimino (2016); Indian J Opthalmol [27]	Retrospective	2006–2014	Italy	7 patients	NA	NA
21	Taki (2017); Ocul Immunol Inflamm [28]	Retrospective	2002–2014	Japan	6 patients	NA	NA
22	Mahajan (2017); Ocul Immunol Inflamm [29]	Retrospective	2004–2015	India	12 patients	NA	NA
23	Kase (2016); Diagn Pathol [30]	Retrospective	2016	Japan	12 patients	Patients with uveitis	4 patients
24	Raja (2016); Retina [31]	Retrospective	2000–2015	United States	25 patients	NA	NA
25	Bonzheim (2015); Blood [32]	Retrospective	2008–2014	Germany	69 patients	NA	NA
26	Pulido (2015); Retina [33]	Retrospective (Case Series)	2015	United States	3 patients	NA	NA
27	Levasseur (2013); JAMA Ophthalmol [34]	Retrospective	1990–2010	Canada	31 patients	NA	NA
28	Wang (2011); Int J Mol Sci [35]	Retrospective	1998–2010	United States	208 patients	NA	NA
29	Ma (2016); Ann Hematol [36]	Retrospective	2003–2013	Taiwan	19 patients	NA	NA
30	Egawa (2015); BMC Ophthalmol [37]	Retrospective	2015	Japan	4 patients	Healthy individuals controlled for age and refractive error	15 patients
31	Wang (2014); Cancer Sci [38]	Retrospective	2005–2011	Japan	33 patients	NA	NA
32	Jang (2013); J Ophthalmic Inflamm Infect [39]	Retrospective	2012–2013	United States	5 patients	NA	NA
33	Teckie (2014); Leuk Lymphoma [40]	Retrospective	1999–2011	United States	18 patients	NA	NA

**Table 2:** Unweighted aggregate data of the different tests. The two highest mean and confidence interval as well as the lowest coefficient of variation values are highlighted with bold.

Test	Cytology	MyD88	PCR IgH Rearrangement	Flow Cytometry	IL10/IL6 > 1
**Mean ± SD**	71% ± 28%	**75%** **±** **14%**	66% ± 29%	58% ± 33%	**86% ± 15%**
**95% CI**	71% ± 2.3%	**75% ± 2.0%**	66% ± 3.5%	58%± 7.5%	**86% ± 1.8%**
**CV**	0.39	**0.19**	0.44	0.57	**0.17**

**Table 3:** Weighted aggregate data of the different tests. The two highest mean and confidence interval as well as the lowest coefficient of variation values are highlighted with bold.

Test	Cytology	MyD88	PCR IgH Rearrangement	Flow Cytometry	IL10/IL6 > 1
**Weighted Mean (wM) ± wSD**	61% ± 27%	**74% ± 11%**	**80% ± 26%**	64% ± 24%	73% ± 17%
**95% CI**	61% ± 2.1%	**74% ± 1.5%**	**80% ± 3.1%**	64% ± 5.4%	73% ± 0.21%
**CV**	0.44	**0.15**	0.32	0.38	**0.23**

## Data Availability

Data are contained within the article or Supplementary Materials.

## Supplementary Materials

The following are available online at www.mdpi.com/article/10.3390/cancers14030598/s1, Table S1: Detailed data of the number of total and positive cytology, *MyD88* and *IgH* Rearrangement samples in the dataset. Table S2: Detailed data of the number of total and positive Flow cytometry and IL10/IL6 Ratio samples in the dataset. Table S3: Calculated sensitivity for all examined methods in the dataset.
