# Supplementary material for: Diagnosing Vitreoretinal Lymphomas—An Analysis of the Sensitivity of Existing Tools
Source: Cancers (Basel). 2022 Jan 25;14(3):598. doi: 10.3390/cancers14030598 (PMC8833443; doi:10.3390/cancers14030598)
Supplement: Supplementary file 1 [file cancers-14-00598-s001.zip › cancers-1532277-supplementary.pdf]

Supplementary Materials

Diagnosing Vitreoretinal Lymphomas—An Analysis of the Sensitivity of Existing Tools

Anahita Sehgal [1], Jose S. Pulido [1,2], Arman Mashayekhi [3], Tatyana Milman [4] and Gabor Gy Deák [5,*]

1 Department of Ophthalmology, Wills Eye Hospital, Sidney Kimmel Medical College, Thomas Jefferson University, Philadelphia, PA 19107, USA; Anahita.Sehgal@students.jefferson.edu (A.S.); jPulido@willseye.org (J.S.P.)
2 Bower Laboratory for Translational Medicine Vickie and Jack Farber, Vision Research Center at Wills Eye Hospital, Wills Eye Hospital, Philadelphia, PA 19107, USA
3 Department of Ophthalmology, Mayo Clinic, Jacksonville, FL 32224, USA; arman_mash@yahoo.com
4 Department of Pathology, Wills Eye Hospital, Sidney Kimmel Medical College, Thomas Jefferson University, Philadelphia, PA 19107, USA; tmilman@willseye.org
5 Department of Ophthalmology, Medical University of Vienna, 1090 Vienna, Austria
* Correspondence: email: gabor.deak@meduniwien.ac.at; Tel.: +43-1-40-4007-9310

Table S1. Detailed data of the number of total and positive cytology, *MyD88* and *IgH* Rearrangement samples in the dataset.

S/N	Study	Number of Cytology Samples	Number of Cytology (+) Samples	Number of MyD88 Samples	Number of MyD88 (+) Samples	Number of PCR *IgH* Rearrangement Samples	Number of PCR *IgH* (+) Samples
1	Giufrrè (2020); Ocul Immunol and Inflamm	33	12	23	19	NA	NA
2	Tsubota (2020); J Clin Med	40	20	NA	NA	NA	NA
3	Arai (2020); Cancer Sci	7	7	7	4	NA	NA
4	Lee (2020); Haematologica	9	3	9	7	9	5
5	Marchese (2019); Br J opthalmol	18	13	NA	NA	NA	NA
6	Shi (2021); Ocul Immunol Inflamm	NA	NA	29	22	NA	NA
7	Tan (2019); Blood	NA	NA	3	3	NA	NA
8	Quintyn (2019); Cytopathology	12	6	8	6	10	5
9	Ito (2019); Graefe's Archive for Clinical and Experimental Ophthalmology	35	27	NA	NA	NA	NA
10	Hoog (2019); Acta Ophthalmol	NA	NA	NA	NA	NA	NA
11	Yonese (2019); Eur J Haematol	17	13	17	12	NA	NA
12	Miserocchi (2019); Retina	12	8	8	8	NA	NA
13	Carreno (2018); Acta Ophthalmol	NA	NA	15	11	7	1
14	Hiemcke-Jiwa (2018); JAMA Ophthal	20	6	21	16	NA	NA
15	Nakahara (2018); BMC Opthalmol	NA	NA	NA	NA	5	4
16	Lee (2019); Retina	22	7	NA	NA	22	19
17	Cho (2018); Ocul Immunol Inflamm	53	6	NA	NA	NA	NA

18	Pochat-Cotilloux (2018); Retina	16	15	NA	NA	NA	NA
19	Cani (2017); Oncotarget	4	2	4	2	NA	NA
20	Cimino (2016); Indian J Opthalmol	7	7	NA	NA	7	7
21	Taki (2017); Ocul Immunol Inflamm	6	6	NA	NA	NA	NA
22	Mahajan (2016); Ocul Immunol Inflamm	10	10	NA	NA	NA	NA
23	Kase (2016); Diagn Pathol	15	14	NA	NA	NA	NA
24	Raja (2016); Retina	NA	NA	23	14	NA	NA
25	Bonzheim (2015); Blood	26	13	26	16	26	17
26	Pulido (2015); Retina	NA	NA	3	2	NA	NA
27	Levasseur (2013); JAMA Ophthalmol	31	27	NA	NA	18	8
28	Wang (2011); Int J Mol Sci	123	66	NA	NA	114	114
29	Ma (2016); Ann Hematol	19	19	NA	NA	NA	NA
30	Egawa (2015); BMC Ophthalmol	3	3	NA	NA	NA	NA
31	Wang (2014); Cancer Sci	28	5	NA	NA	28	28
32	Jang (2013); J Ophthalmic Inflamm Infect	5	5	NA	NA	NA	NA
33	Teckie (2014); Leuk Lymphoma	18	17	NA	NA	NA	NA

Table S2. Detailed data of the number of total and positive Flow cytometry and IL10/IL6 Ratio samples in the dataset.

S/N	Study	Number of Flow Cytometry Samples	Number of Flow (+) Samples	Number of IL 10/IL 6 Ratio Samples	Number of IL10/IL6 Ratio (+) Samples
1	Giufrrè (2020); Ocul Immunol and Inflamm	NA	NA	NA	NA
2	Tsubota (2020); J Clin Med	NA	NA	40	32
3	Arai (2020); Cancer Sci	NA	NA	7	7
4	Lee (2020); Haematologica	NA	NA	9	9
5	Marchese (2019); Br J opthalmol	12	8	NA	NA
6	Shi (2021); Ocul Immunol Inflamm	NA	NA	NA	NA
7	Tan (2019); Blood	NA	NA	NA	NA
8	Quintyn (2019); Cytopathology	NA	NA	10	8
9	Ito (2019); Graefe's Archive for Clinical and Experimental Ophthalmology	NA	NA	NA	NA
10	Hoog (2019); Acta Ophthalmol	10	6	10	9
11	Yonese (2019); Eur J Haematol	17	9	17	16
12	Miserocchi (2019); Retina	NA	NA	NA	NA
13	Carreno (2018); Acta Ophthalmol	NA	NA	NA	NA
14	Hiemcke-Jiwa (2018); JAMA Ophthal	17	10	NA	NA
15	Nakahara (2018); BMC Opthalmol	5	0	NA	NA
16	Lee (2019); Retina	NA	NA	22	18
17	Cho (2018); Ocul Immunol Inflamm	NA	NA	NA	NA
18	Pochat-Cotilloux (2018); Retina	NA	NA	16	15

19	Cani (2017); Oncotarget	NA	NA	NA	NA
20	Cimino (2016); Indian J Opthalmol	NA	NA	NA	NA
21	Taki (2017); Ocul Immunol Inflamm	NA	NA	NA	NA
22	Mahajan (2016); Ocul Immunol Inflamm	NA	NA	NA	NA
23	Kase (2016); Diagn Pathol	NA	NA	16	10
24	Raja (2016); Retina	NA	NA	NA	NA
25	Bonzheim (2015); Blood	NA	NA	NA	NA
26	Pulido (2015); Retina	NA	NA	NA	NA
27	Levasseur (2013); JAMA Ophthalmol	NA	NA	NA	NA
28	Wang (2011); Int J Mol Sci	NA	NA	98	53
29	Ma (2016); Ann Hematol	14	14	NA	NA
30	Egawa (2015); BMC Ophthalmol	NA	NA	4	4
31	Wang (2014); Cancer Sci	NA	NA	NA	NA
32	Jang (2013); J Ophthalmic Inflamm Infect	NA	NA	NA	NA
33	Teckie (2014); Leuk Lymphoma	NA	NA	NA	NA

Table S3. Calculated sensitivity for all examined methods in the dataset.

S/N	Study	Cytology Sensitivity	MyD88 Sensitivity	PCR IgH Rearrangement Sensitivity	Flow Sensitivity	IL10/IL6 Sensitivity
1	Giufrrè (2020); Ocul Immunol and Inflamm	0.36	0.83	NA	NA	NA
2	Tsubota (2020); J Clin Med	0.50	NA	0.35	NA	0.80
3	Arai (2020); Cancer Sci	1.00	0.67	NA	NA	1.00
4	Lee (2020); Haematologica	0.33	0.78	0.56	NA	1.00
5	Marchese(2019); Br J opthalmol	0.72	NA	NA	0.75	NA
6	Shi (2021); Ocul Immunol Inflamm	NA	0.85	NA	NA	NA
7	Tan (2019); Blood	NA	1.00	NA	NA	NA
8	Quintyn (2019); Cytopathology	0.50	0.75	0.50	NA	0.80
9	Ito (2019); Graefe's Archive for Clinical and Experimental Ophthalmology	0.77	NA	NA	NA	
10	Hoog (2019); Acta Ophthalmol	NA	NA	NA	0.60	0.90
11	Yonese (2019); Eur J Haematol	0.76	0.71	NA	0.53	0.94
12	Miserocchi (2019); Retina	0.75	1.00	NA	NA	NA
13	Carreno (2018); Acta Ophthalmol	NA	0.73	0.14	NA	NA
14	Hiemcke-Jiwa (2018); JAMA Ophthal	0.30	0.75	NA	0.59	NA
15	Nakahara (2018); BMC Opthalmol	NA	NA	0.80	0	NA
16	Lee (2019); Retina	0.32	NA	0.86	NA	0.82
17	Cho (2018); Ocul Immunol Inflamm	0.11	NA	NA	NA	NA
18	Pochat-Cotilloux (2018); Retina	0.94	NA	NA	NA	0.94
19	Cani (2017); Oncotarget	0.50	0.50	NA	NA	NA
20	Cimino (2016); Indian J Opthalmol	1.00	NA	1.00	NA	NA
21	Taki (2017); Ocul Immunol Inflamm	1.00	NA	NA	NA	NA
22	Mahajan (2016); Ocul Immunol	1.00	NA	NA	NA	NA

	Inflamm					
23	Kase (2016); Diagn Pathol	0.93	NA	NA	NA	0.67
24	Raja (2016); Retina	NA	0.61	NA	NA	NA
25	Bonzheim (2015); Blood	0.50	0.62	0.65	NA	NA
26	Pulido (2015); Retina	NA	0.67	NA	NA	NA
27	Levasseur (2013); JAMA Ophthalmol	0.87	NA	0.45	NA	NA
28	Wang (2011); Int J Mol Sci	0.54	NA	1.00	NA	0.54
29	Ma (2016); Ann Hematol	1.00	NA	NA	1.00	NA
30	Egawa (2015); BMC Ophthalmol	1.00	NA	NA	NA	1.00
31	Wang (2014); Cancer Sci	0.89	NA	1.00	NA	NA
32	Jang (2013); J Ophthalmic Inflamm Infect	1.00	NA	NA	NA	NA
33	Teckie (2014); Leuk Lymphoma	0.94	NA	NA	NA	NA
